# The factor structure of a short acculturation scale for Filipino Americans in an adult U.S.‐born sample

**DOI:** 10.1002/jcop.21955

**Published:** 2018-05-21

**Authors:** Felicitas A. dela Cruz, Chong Ho Yu, Kristine I. Vindua

**Affiliations:** ^1^ Azusa Pacific University

## Abstract

The influx of non‐European immigrants since 1965 ushered the development and use of acculturation measures in immigrant health studies. A Short Acculturation Scale for Filipino Americans (ASASFA) represents a validated, unidirectional ethnic‐specific measure used with first‐generation FAs. ASASFA's psychometric properties with adult U.S.‐born children—the second generation—remain unexplored. This study determined (a) the factor structure of ASASFA with adult U.S.‐born FAs and (b) the predictors of their acculturation scores. A secondary analysis was conducted on ASASFA data from a mental health survey of 116 U.S.‐born FAs. Exploratory factor and parallel analyses showed a two‐factor solution: language use and preference (Factor 1) and ethnic social relations (Factor 2). Ordinary least squares regression indicated gender and ethnic self‐identification predict Factor 1 scores; self‐identification solely predicts Factor 2 scores. Results demonstrate ASASFA's validity and parsimony, supporting its use in FA health studies when lengthy bidirectional acculturation measures become impractical.

## 1 INTRODUCTION

In contrast to the late 19^th^‐ and early 20^th^‐century immigration waves of Europeans to the United States, the 1965 Naturalization and Immigration law ushered the influx of new immigrants primarily from Latin America, the Caribbean, and Asia, transforming the United States into a more diverse multicultural and multiethnic society (Grieco et al., 2012; Pew Research Center, 2013; Portes & Rumbaut, 2006). Upon arrival, these new U.S. immigrants undergo acculturation—the “process by which an immigrant adopts the language, customs, behaviors, and attitudes of the host culture” (Lee, O'Neill, Ihara, & Chae, 2013, p. 2). Acculturation has been shown to influence dietary practices (dela Cruz, Lao, & Heinlein, 2013; Serafica, Lane, & Ceria‐Ulep, 2013); medication adherence, lifestyle behaviors, and blood pressure control (Tailakh et al., 2014); and obesity and cardiovascular risk factors (Shah et al., 2015).

Although the process of acculturation begins with new immigrants, the acculturation of their U.S.‐born children—the second generation—bears more long‐term effects on American society (Portes & Rumbaut, 2005). Currently, 20 million U.S.‐born children of the new immigrants have come of age (Pew Research Center, 2013). As U.S. citizens, their acculturation patterns and adaptation lay the foundation for the enduring character of their ethnic communities (Portes & Rumbaut, 2005).

The arrival of the new immigrants spawned the development of a variety of acculturation measures (Balls Organista, Marin, & Chung, 2010), with most of these measures focusing on Latin American acculturation (Gamst, Liang, & Der‐Karabetian, 2011). Asians, a heterogeneous racial group, now comprise the fastest growing immigrant population in the United States (Malik, 2015, May 21); nevertheless, a limited number of ethnic‐specific acculturation measures exist (Gamst, Liang, & Der‐Karbetian, 2011). Moreover, research on the acculturation of their adult U.S.‐born children is scarce due to the paucity of psychometrically validated ethnic‐specific measures.

Filipinos presently represent the fourth largest immigrant group by country of origin behind Mexico, China, and India (McNamara & Batalova, 2015). Post‐1965 Filipino migration features a greater proportion of highly educated professionals such as physicians, nurses, and engineers. The U.S. Census Bureau (2015) estimates more than 3.6 million Filipino Americans (FAs) in the United States who reported their ancestry as Filipino alone or in combination with one or more racial groups, with the largest number residing in the greater Los Angeles metropolitan area (McNamara & Batalova, 2015). U.S.‐born FAs account for 1.23 million of the U.S. population (Migration Policy Institute, 2014).

The influx of Filipinos to the United States as the desired host country stems from the U.S. colonization of the Philippines from 1898–1946, which differentiates Filipinos from other Asian immigrants. The three centuries of Spanish rule prior to U.S. colonization further differentiates Filipino immigrants. The U.S. colonization of the Philippines exposed Filipinos to American culture. This colonization established English as the medium of instruction and official language along with F*ilipino* or *Tagalog*, the national language. The United States maintained a continued military presence until 1994, well after Philippine independence was granted in 1946, when all military bases were handed over to the Philippines (Migration Policy Institute, 2014). Despite this exposure to American culture, Filipino immigrants still undergo acculturation upon arrival in the United States.

On the other hand, the children of Filipino immigrants who are born in the United States live in two contrasting cultures: the American culture that emphasizes individualism and autonomy, and the Philippine culture that underscores collectivism—the primacy of family and harmonious relations (Nadal, 2011). They experience the American mainstream culture in their communities, schools, and work places, and through the media, and the Philippine culture at home with parents and extended families as well as during the observance of and participation in Filipino cultural events. Therefore, U.S.‐born FAs undergo acculturation in two disparate cultures. They navigate and adapt variously to both cultures through the process of acculturation, especially because these two cultures manifest contrasting orientations. Given the relationship between acculturation and health outcomes, and the variability of acculturation across individuals and generations, an appropriate measure of acculturation becomes an invaluable tool when designing culturally‐tailored health care interventions for U.S.‐born FAs.

A Short Acculturation Scale for Filipino Americans (ASASFA; dela Cruz, Padilla, & Agustin, 2000; dela Cruz, Padilla, & Butts, 1998) represents the only ethnic‐specific acculturation measure and hence the most used for FAs. Since its development and original validation, the studies using ASASFA involved first‐generation (Philippine‐born) Filipinos (dela Cruz & Galang, 2008; dela Cruz et al., 2013; Ea, 2007; Kataoka‐Yahiro, 2010; McAdam, Stotts, Padilla, & Puntillo, 2005; Reyes & Cohen, 2013; Serafica et al., 2013). Very little research on its use with U.S.‐born FAs exists. The purpose of this study therefore was to fill this gap in the literature. Specifically, it sought to determine (a) the factor structure of the scale with adult U.S.‐born FAs and (b) the predictors of their acculturation scores.

### 1.1 ASASFA

ASASFA underwent its initial validation in 1998 (dela Cruz et al., 1998). The measure traces its development and adaptation from the Short Acculturation Scale for Hispanics (SASH; Marin, Sabogal, Marin, Otero‐Sabogal, & Perez‐Stable, 1987). ASASFA has two validated language versions—in English and in F*ilipino* or *Tagalog*, the Philippine national language. These language versions underwent steps to ensure their content, technical, experiential, semantic, and conceptual equivalence (dela Cruz et al., 2000). Consistent with SASH (Marin et al.,^1987^), the measures exclude sociodemographics in the scales to maintain their use as correlates of acculturation.

In the initial study, principal components analysis revealed three dimensions being measured by the scale in each language version: language use and preference at home, work, and with friends; language use and preference in media—TV, film, and radio; and preference for ethnic social relations. Each language version obtained an overall Cronbach's alpha coefficient of .85, indicating high internal consistency among the items (dela Cruz et al., 1998, 2000). Studies that have used this measure have shown appropriate coefficient alphas as evidence of its reliability and have been consistent with the theoretical literature (Ayers, Atkins, & Lee, 2010).

The original validation study showed that ethnic identity, food preference, educational level, length of U.S. residence, family income, and age on arrival to the United States were associated with the acculturation level of first‐generation FAs. Stepwise multiple regression indicated that ethnic identification serves as the primary factor associated with their acculturation level (dela Cruz et al., 1998, 2000).

ASASFA represents a unidirectional but multidimensional model of acculturation (Flannery, Riese, & Yu, 2001). This model posits that acculturation occurs in a continuum—starting with total immersion in the heritage culture, progressing to biculturalism, and finally assimilation into the host culture (Abe‐Kim, Okazaki, & Goto, 2001; Zhang & Tsai, 2014).

Another acculturation model, the bidirectional model, contrasts with the unidirectional model. The bidirectional model proposes that the simultaneous acquiring or adhering to a new culture and maintaining the heritage culture represent two independent processes (Abe‐Kim et al., 2001; Zhang & Tsai, 2014). Accordingly, U.S.‐born FAs concurrently learn and selectively acquire and adapt the customs, behaviors, and attitudes of their heritage culture along with the American mainstream culture.

Despite the increasing use of bidirectional measures, a unidirectional measure such as ASASFA provides an economical and parsimonious tool (Flannery, Reise, & Yu, 2001) for health community surveys that include acculturation as a variable. Furthermore, different models may apply to different subgroups (Zhang & Tsai, 2014). In a previous study, the unidimensional model applied more to Chinese Americans who came to the United States after adolescence, while the bidirectional model applied more to Chinese Americans who immigrated prior to adolescence (Tsai, Ying, & Lee, 2000; Zhang & Tsai, 2014).

## 2 METHOD

### 2.1 Research design and sample

A secondary analysis was conducted on the acculturation data collected from 116 U.S.‐born FAs, as part of a survey that investigated the factors that relate to and predict the mental health of Philippine‐born and U.S.‐born FAs (Vindua, 2010). The sample was recruited from local organizations in the Greater Los Angeles Area. Study participants met at a designated location, either one on one or in small groups of 5–10 individuals, where they completed the survey questionnaires. The study received the approval of the university's institutional review board.

### 2.2 Measures

This study used two measures from the original survey as follows.

#### 2.2.1 Sociodemographic questionnaire

A sociodemographic questionnaire obtained information regarding (a) age, (b) gender, (c) marital status, (d) educational level, (e) occupation, (f) occupational status, (g) household income, (h) religious preference, (i) food preference, and (j) self‐identification.

#### 2.2.2 ASASFA

ASASFA assessed language use and preference at work and home and with friends; and language use and preference for media. The measure uses a scale of 1 = only Philippine languages, 2 = more Philippine languages than English, 3 = both equally, 4 = more English than Philippine languages, and 5 = only English. To assess preference for ethnic social relations, the measure uses a scale of 1 = all Filipinos, 2 = more Filipinos than Americans, 3 = about half and half, 4 = more Americans than Filipinos, and 5 = All Americans (dela Cruz et al., 1998, 2000). Based on the value assigned to the response, the total acculturation score ranged from 12 to 60; the mean score for the total scale and subscales ranged from 1 to 5. Lower scores reflect lower acculturation levels toward the American culture and middle scores signal biculturalism, while higher scores indicate higher acculturation level toward the American culture. This study on U.S.‐born FAs focuses only on the English version of the scale.

### 2.3 Data analysis

Descriptive statistics were used to examine the sociodemographic characteristics of the study participants and their responses to ASASFA, using SPSS (version 22). In addition, we computed the Kaiser‐Meyer‐Olkin measure of sampling adequacy and Bartlett's test of sphericity to determine the factorability of the data (Young & Pearce, 2013). We employed exploratory factor analysis (EFA) to explore the scale's factor structure and to generate the loading plot, using JMP (pro version 13).

To select the number of factors in EFA, we initially viewed the inflection point in the scree plot; in addition, we adopted the loading plot approach and parallel analysis for verification. A loading plot is a graphical technique to depict the direction, proximity, and grouping pattern of the variables (the scale items). The relationship between variables and factors can be expressed as weighted linear equations, which can be re‐expressed as vectors in the loading plot (Jacoby, 1998; SAS Institute, 2016; Yan & Kang, 2003).

As the name implies, EFA is exploratory in nature, and thus it is error‐prone (Osborne, 2014). As a remedy, many authors recommend using parallel analysis to verify the factor structure (Buja & Eyuboglu, 1992; Glorfeld, 1995; Ledesma & Valero‐Mora, 2007). Parallel analysis is a form of resampling, such that the existing sample is regarded as a pseudo‐population (Yu, Osborn‐Popp, DiGangi, & Jannasch‐Pennell, 2007). The algorithm generates a set of random data correlation matrices by resampling from the sample, and then the average eigenvalues and the 95th percentile eigenvalues are computed. The underlying logic is: the extracted factors must substantively outperform the random factors by chance alone. We used SAS (version 9.4) as the programming environment for running the parallel analysis macros developed by O'Connor (2000). In this study 1,000 re‐samples were used in parallel analysis.

Additionally, the relationships between acculturation and certain sociodemographic variables were examined after the factor structure was ascertained. Ordinary least squares (OLS) regression was employed to examine which of these demographic variables—age, gender, educational level, occupational status, household income, religious preference, food preference, and self‐identification—are predictors of the acculturation subscales. Marital status was excluded because most participants were single. Likewise, occupation was omitted because 44.8% of the sample left this item unanswered; these study participants were looking for work or studying full time.

To avoid estimating too many parameters and yielding an unstable model due to lack of degrees of freedom, we combined the following demographic variables.

- Age into two classes: young adults (18–34 years) and middle/older adults (35–65 years)
- Educational level into two groups: some college and college graduate
- Household income into three categories (Hidalgo et al., 2013): low income (< $5–29,000), middle income ($30–75,000), and high income (> $75,000)
- Religious preference into two sets because most Filipinos are Catholic: Catholic and non‐Catholic
- Food preference into three clusters: exclusively Filipino and mostly Filipino food with some American food to Filipino food, about equally Filipino and American food, and mostly American with some Filipino food and exclusively American food to American food
- Self‐identification responses into three groups: very Filipino and more Filipino than American to Filipino, almost 50/50 Filipino and American, and more American than Filipino and very American to American

The American Psychological Association Task Force on Statistical Inference (1996; Wilkinson and the Task Force on Statistical Inference, 1999) recommended routine use of confidence intervals (CIs) in the presentation of statistical results. Consonant with this recommendation, the crucial predictors that OLS identified were examined by utilizing CIs and their graphical representation—the diamond plot. The bar inside each diamond on the graph is the group mean whereas the upper point and the lower point of the diamonds are the upper bound and the lower bound of the CI, respectively. The group difference is detected by whether the CIs of the groups overlap or not.

## 3 RESULTS

### 3.1 Sociodemographic characteristics of sample

Table 1 shows the sociodemographic characteristics of the sample. Most of the U.S.‐born sample comprised primarily of young adults with a mean age of 30 years (standard deviation [*SD*] = 11.18), females, single (never married), with some college education. The majority revealed a household income of > $75,000. The sociodemographic characteristics of age, marital status, level of education, and household income correspond to the Pew Research Center's (2013) portrait of Asian American second generation. Nearly two thirds of the U.S.‐born FA sample reported their religious preference as Catholic. Most of the sample designated their food preference as equally Filipino and American; however, equal proportions (20.7%) of the sample preferred either mostly Filipino than American food, or mostly American than Filipino food. For self‐identification, the majority specified almost 50/50 Filipino and American, while nearly a fourth and slightly over a fifth of the sample identified themselves as more Filipino than American and more American than Filipino, respectively.

**Table 1:** Sociodemographic characteristics of U.S.‐born study participants (N = 116)

Characteristic	Frequency	Percent
Age: Mean, SD (30,11.18)		
Young adults (18‐34)	82	70.7
Middle adults (35‐54)	27	23.3
Older adults (55‐65)	7	6.0
Gender:		
Male	38	32.8
Female	78	67.2
Marital Status:		
Married	6	5.2
Single, never married	102	87.9
Single, living with someone	8	6.9
Educational level:		
Some college	80	69
College graduate	18	15.5
Some graduate school	10	8.6
Master's degree	6	5.2
Doctorate	2	1.7
Occupation:		
Professionals/technicians	20	17.2
Administrative support	26	22.4
Sales workers	6	5.2
Operatives	2	1.7
Service workers	10	8.6
None/not provided	52	44.8
Occupational status:		
Full‐time	26	22.4
Part‐time	38	32.8
Looking for work	16	13.8
Studying full‐time	36	31.0
Household income:		
< $5,000	8	6.9
$5‐10,000	2	1.7
$11‐20,000	2	1.7
$21‐30,000	0	0
$31‐40,000	8	6.9
$41‐50,000	12	10.3
$51‐75,000	34	29.3
>$75,000	48	41.4
Welfare benefits (SSI, WIC, etc.)	2	1.7
Religious preference:		
Catholic	73	62.9
Protestant	22	19
Seventh Day Adventist	2	1.7
*Iglesia ni Cristo* (Church of Christ)	2	1.7
Agnostic/Atheist	4	3.4
No preference	13	11.2
Food preference:		
Exclusively Filipino	0	0
Mostly Filipino, some American	24	20.7
About equally Filipino and American	66	56.9
Mostly American, some Filipino	24	20.7
Exclusively American	2	1.7
Self‐identification:		
Very Filipino	10	8.6
More Filipino than American	28	24.1
Almost 50/50 Filipino and American	48	41.4
More American than Filipino	24	20.7
Very American	6	5.2

*Note*. SD = standard deviation; SSI = Supplemental Security Income; WIC = Special Supplemental Program for Women, Infants, and Children.

### 3.2 Preliminary analysis

While computing the descriptive statistics of the responses to the ASASFA, we found that the respondents uniformly answered “All English” to item AC7, which asked, “In what language(s) are the radio programs you usually listen to?” Because of this lack of variability, we deleted this item in all subsequent statistical computations.

The sample's mean total acculturation score registered at 3.91 (*SD *= .44). The mean scores for the items on language use and preference (AC1‐6 and 8) and ethnic social relations (SR9‐12) measured at 4.56 (*SD *= .52) and at 2.78 (*SD *= .67), respectively. The English language use and preference mean scores showed that (a) those who identified themselves as more American than Filipino indicated the highest scores (4.78, SD = .54); (b) those who identified themselves as almost 50/50 Filipino and American and those who claimed themselves as more Filipino than American showed scores of 4.63 (SD = .27) and 4.36 (SD = .46), respectively; and (c) those who identified themselves as very Filipino showed the lowest scores (3.96, SD = .91). Nobody indicated a preference for and use of only the English language.

In contrast, in ethnic social relations, those who identified themselves as very Filipino, more Filipino than American, and almost 50/50 Filipino and American showed preference for Filipinos, with mean scores registering at 2.5 (SD = .39), 2.61 (SD = .73), and 2.68 (SD = .56), respectively. On the other hand, those who identified themselves as more American than Filipino and very American indicated mean scores in ethnic social relations ranging from 3.01 (SD = .42) to 3.83 (SD = < 1.008) respectively.

Preliminary analysis revealed that ASASFA data met the requirements for factor analysis: (a) the Kaiser‐Meyer‐Olkin measure yielded .758, which meets the cutoff above .50, and (b) the Bartlett's test of sphericity produced significant results (approximate X^2^ = 820.741, degree of freedom = 55, *p* < .0001), to confirm that the variables have patterned relationships (Young & Pearce, 2014).

### 3.3 EFA

**Figure 1:**
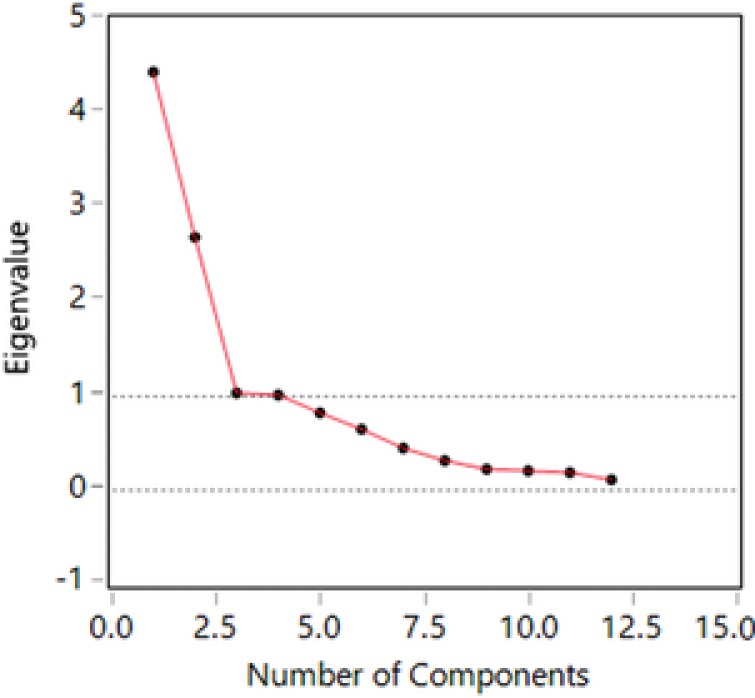
Scree plot for A Short Acculturation Scale for Filipino Americans

EFA suggested a two‐factor solution, based on the inflection point of the scree plot (Figure 1).

Table 2 presents the factor loading output by varimax rotation in EFA and communality of each variable. Believing that the two factors (constructs) are independent (orthogonal), we adopted the varimax method. The results are clear‐cut. Variables that are assigned to Factor 1 have factor loadings ranging from .42 to .89, whereas variables belonging to Factor 2 have loadings between .60 and .90. The communality of a particular variable is the proportion of variation in that variable explained by the factors. For example, if AC3 (language[s] spoken at home) is used to regress against the two proposed factors, then about 83% of the variation in AC3 can be explained by the factor model.

**Table 2:** Summary of items and factor loadings of ASASFA using exploratory factor analysis with varimax rotation and item communality

	Factor loading		h^2^
Item	1	2	
AC3 Language(s) spoken at home	**.89**	.17	.83
AC1 Language(s) read and spoken	**.88**	.12	.79
AC5 Language(s) spoken with friends	**.81**	−.02	.66
AC8 Language(s) of preferred movies, TV, and radio programs	**.70**	.07	.50
AC4 Language(s) used with thinking	**.66**	−.09	.45
AC2 Language(s) spoken as a child	**.63**	.18	.43
AC6 Language(s) of TV programs usually watched	**.42**	.07	.18
SR11 Ethnicity of visitors or persons visited	.10	**.90**	.82
SR9 Ethnicity of close friends	.07	**.88**	.78
SR10 Ethnicity of social gatherings	.03	**.67**	.45
SR12 Ethnicity of children's friends	.09	**.60**	.37

*Note*. ASASFA = A Short Acculturation Scale for Filipino Americans.

Boldface indicates highest factor loadings.

Based on the EFA results, we retained the labels of the two constructs: language use and preference to represent Factor 1 and ethnic social relations to represent Factor 2. The Cronbach's alpha of Factor 1 and Factor 2 is .86 and .81, respectively, and the total scale reliability is .82.

**Figure 2:**
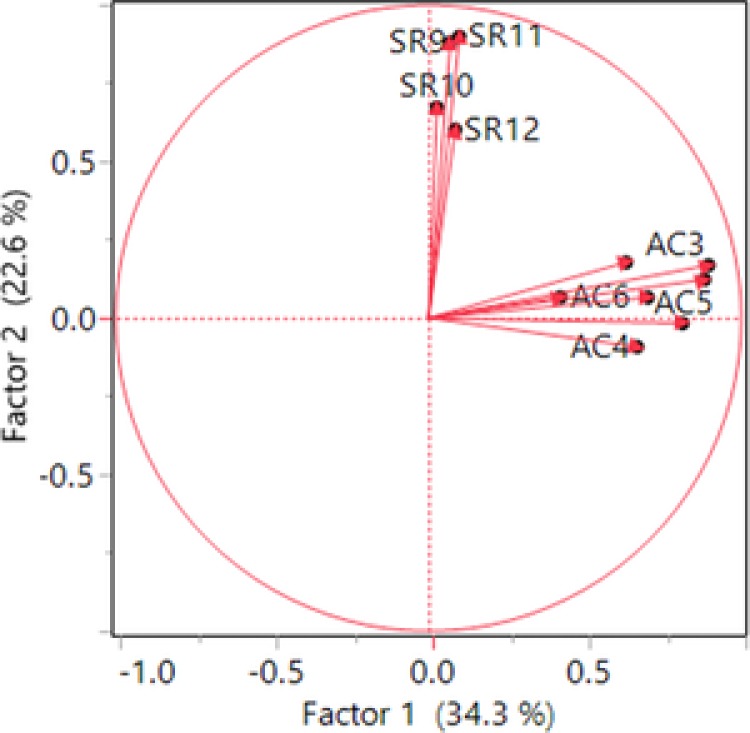
Loading plot of vectors of the variables for Factor 1 and Factor 2

The loading plot (Figure 2) provides further evidence of ASASFA's factor structure. The vectors that represent the variables form two clusters. The proximity of all language use and preference (AC) items is close as are all ethnic social relations (SR) items. Although the two sets are not perfectly orthogonal (90 degrees), they are far apart from each other, and thus the two constructs should be considered distinct.

Parallel analysis provides additional evidence. Table 3 shows the original eigenvalues, the means of resampled eigenvalues, and the 95^th^ percentile of resampled eigenvalues. A two‐factor solution based on the original eigenvalues can substantively outperform the random results.

**Table 3:** Results of parallel analysis

Number of factors	Original eigenvalues	Means of resampled eigenvalues	95th percentile
**1**	**4.12**	**0.63**	**0.80**
**2**	**2.35**	**0.46**	**0.59**
3	0.66	0.34	0.44
4	0.42	0.23	0.31
5	0.15	0.13	0.22
6	0.01	0.05	0.11
7	−0.03	−0.03	0.03
8	−0.07	−0.11	−0.05
9	−0.13	−0.18	−0.13
10	−0.15	−0.25	−0.20
11	−0.18	−0.33	−0.28

### 3.4 Predictors of acculturation scores

#### 3.4.1 Language use and preference scores

**Figure 3:**
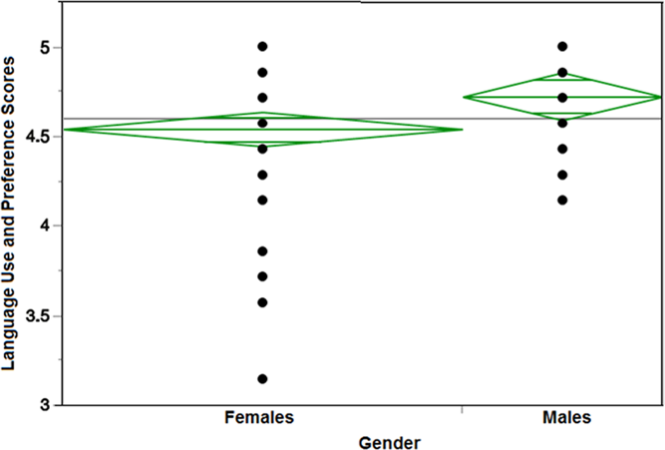
Diamond plots comparing females and males in relation to language use and preference scores

**Figure 4:**
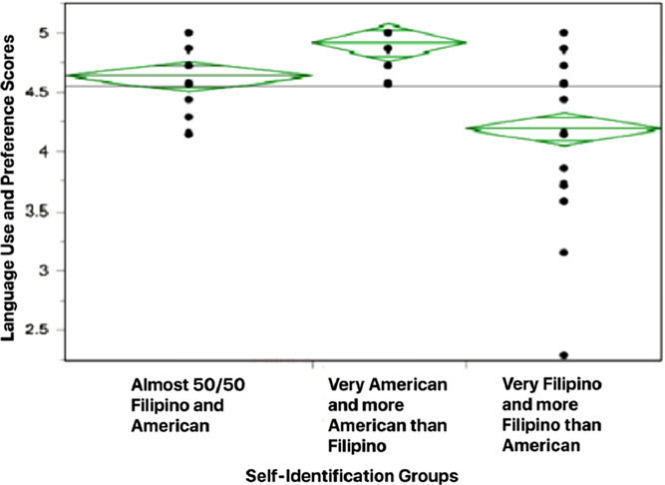
Diamond plots comparing self‐identification groups in relation to language use and preference scores

OLS regression indicates that gender *(p = 0.0016)* and self‐identification *(p <* *.0001)* were crucial predictors of language scores (Table 4). The diamond plots show that males have a significantly higher score than females after two female outliers were removed (Figure 3) and Figure 4 compares the self‐identification groups in relation to language use and preference scores. All the diamond plots do not overlap, indicating that all three self‐identification groups significantly differ from each other. The CI information on gender can be found at Table 5 and on self‐identification at Table 6.

**Table 4:** Predictors of language use and preference scores

Variable	*df*	Sum of squares	*F* Ratio	*p*
Age	1	0.022	0.14	0.7096
Gender	1	1.67	10.52	0.0016*
Household income	2	0.65	2.04	0.1352
Food preference	2	0.69	2.19	0.1173
Occupational status	3	0.71	1.50	0.2198
Religious preference	1	0.11	0.67	0.4139
Educational level	1	0.05	0.33	0.5671
Self‐identification	2	6.45	20.34	<.0001*

*Note*. df = degrees of freedom.

*Significance.

**Table 5:** Confidence intervals for gender in relation to language use and preference scores

Gender	n	Mean	*SE*	Lower 95%	Upper 95%
Female	76	4.54	0.05	4.44	4.63
Male	38	4.72	0.07	4.59	4.86

*Note*. SE = standard error.

**Table 6:** Confidence intervals for self‐identification groups in relation to language use and preference scores

Self‐identification groups	n	Mean	*SE*	Lower 95%	Upper 95%
Almost 50/50 Filipino and American	48	4.63	0.06	4.51	4.76
Very American and More American than Filipino	30	4.91	0.08	4.76	5.07
Very Filipino and more Filipino than American	38	4.19	0.07	4.05	4.33

*Note*. SE = standard error.

#### 3.4.2 Ethnic social relations scores

**Figure 5:**
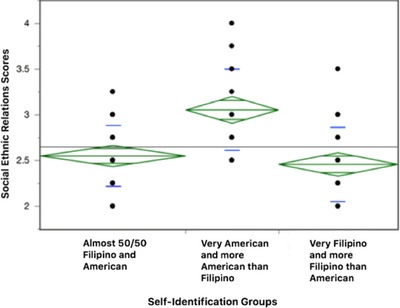
Diamond plots comparing self‐identification groups in relation to ethnic social relations scores

Another OLS regression model shows that only self‐identification (Table 7) was a crucial predictor of social ethnic relation scores *(p = .0047)*. After eight outliers were removed, the diamond plot, as displayed in Figure 5, indicated that there was no significant difference between “almost 50/50 Filipino and American” and “very Filipino and more Filipino than American,” but the social ethnic relation score of “very American and more American than Filipino” is significantly higher than the other two groups. The CI information is shown in Table 8.

**Table 7:** Predictors of social ethnic relations scores

Predictor variables	*df*	Sum of squares	*F* Ratio	*p*
Age	1	0.07	0.18	0.6744
Gender	1	0.28	0.72	0.3979
Household income	2	0.29	0.38	0.6836
Food preference	2	0.14	0.18	0.8371
Occupational status	3	1.13	0.98	0.4045
Religious preference	1	0.95	2.47	0.1194
Educational level	1	0.02	0.06	0.8074
Self‐identification	2	4.34	5.65	0.0047*

*Note*. df = degrees of freedom.

^*^Significance.

**Table 8:** Confidence intervals for self‐identification groups in relation to social ethnic relations scores

Self‐identified groups	n	Mean	SE	Lower 95%	Upper 95%
Almost 50/50 Filipino and American	44	2.5	0.06	2.43	2.6676
Very American and more American than Filipino	28	3.05	0.07	2.91	3.1995
Very Filipino and more Filipino than American	36	2.46	0.06	2.33	2.5871

*Note*. SE = standard error.

## 4 DISCUSSION

This study examined the factor structure of ASASFA in adult U.S.‐born FAs as well as the sociodemographic predictors of their acculturation scores. EFA revealed that the scale measured two factors—language use and preference and ethnic social relations, with the deletion of one item related to the language of radio programs listened to. Parallel analysis supported the scale's factor structure. OLS regression showed that gender and ethnic self‐identification predicted language use and preference scores of U.S.‐born FAs. Similarly, OLS regression identified ethnic self‐identification as the sole predictor of ethnic social relations scores.

The total mean acculturation score (3.91, SD = .44) of adult U.S.‐born FAs indicates an acculturation toward the American culture, especially with their high mean score (4.56, SD = .52) on preference for and use of the English language. However, the mean score for ethnic social relations (2.78, SD = .67) signifies a stronger attachment to members of the heritage culture. This biculturalism highlights the salience of mainstream American societal socialization outside the home and of family socialization to the heritage culture at home. The results also underscore the continuing acculturation of U.S.‐born FA adults to both cultures that they grew up in (Schimmele & Wu, 2015). The finding on ethnic self‐identification as a crucial predictor of the acculturation of U.S.‐born FAs concurs with the findings of the original validation of ASASFA with Philippine‐born immigrants (dela Cruz et al., 1998, 2000).

### 4.1 Factor structure of ASASFA

The factor structure of ASASFA in adult U.S.‐born FAs dovetails with the two most common dimensions measured by major ethnic‐specific acculturation measures—language use and preference, and preference in social ethnic relations (Celenk & Van de Vijer, 2011; Matsudaira, 2006; Zane & Mak, 2003). Being born of parents speaking a language at home that differs from the mainstream American language used outside of the home, U.S.‐born FAs reveal a shift to English language use and preference as a key aspect of their acculturation to American society. At the same time, they disclosed a preference for co‐ethnic Filipinos in their ethnic social relations. Given the two contrasting cultures that U.S.‐born FAs confront every day, these two dimensional results signify biculturalism—the key anchors of their acculturation to the American culture and to the Philippine culture.

With the deletion of the item related to the language of radio programs listened to, ASASFA for U.S.‐born FAs now comprises 11 items; this short scale falls within the mean of 11 items of most acculturation measures (Celenk & Van de Vijer, 2011). The total scale reliability (.82) and dimensional reliabilities of language use and preference (.86) and ethnic social relations (.81) all meet the minimum standard of .80 (Nunnally & Bernstein, 1994). The factor structure and reliabilities of ASASFA indicate the robustness of its psychometric properties, suggesting its utility as a parsimonious acculturation measure for U.S.‐born FAs.

### 4.2 Sociodemographic predictors of acculturation scores

#### 4.2.1 Language use and preference

Using OLS regression, our study revealed that U.S.‐born FA males indicated a preference for and use of the English language compared to U.S.‐born FA females. This finding can be attributed to the practice of the traditional Filipino immigrant family: to keep a more sheltered life for daughters than sons. Consequently, daughters are expected to stay more at home with members of the family to imbibe their role in maintaining family ties and cultural traditions—activities that foster the use and maintenance of the heritage language (Espiritu, 2001). Our results concur with similar findings in an earlier study that revealed U.S.‐born female students were more competent in the heritage language of their immigrant parents (Portes & Hao, 1998).

In addition, data showed that all U.S.‐born FAs clearly use and prefer the English language, highlighting the reality of its instrumental use in navigating day‐to‐day life in the United States. But the strength and intensity of this acculturative attitude and behavior varied according to their disclosed ethnic identity. The incremental increase in the English language use and preference mean scores, from those who claimed their identity as Filipinos, to 50/50 Filipinos and Americans, and to Americans, support the strength of this attitude and behavior. Likewise, the significant differences in the scores between these ethnic identification groups reinforce their language inclinations. These results align with previous studies that found a strong link between ethnic identification and language use and preference—that these aspects of acculturation entwine with each other (Phinney, Romero, Nava, & Huang, 2001; Portes & Schauffer, 1994).

#### 4.2.2 Ethnic social relations

OLS regression further revealed the significant role ethnic identity plays among adult U.S.‐born FAs’ preference for ethnic social relations. On the whole, based on their ethnic identity, their ethnic social relations mean scores were lower along the acculturation scale continuum, compared to their language use and preference mean scores. The ethnic social relations mean scores gradually increased from those identifying themselves as Filipinos to those who identified themselves as Americans.

As in language use and preference for English, significant differences appeared in the preferences for ethnic social relations based on professed ethnic self‐identity. As indicated by the diamond plots in Figure 5, those who identified themselves very Filipino and more Filipino than American as well as those who classified themselves as almost 50/50 Filipino and American preferred co‐Filipino Americans in social relations more than those who categorized themselves as more American than Filipino and very American. Thus, our study results capture the within‐group acculturation differences among U.S.‐born FAs based on their ethnic self‐identification.

However, even the higher social ethnic relations mean scores of those who claimed more American identity still maintain ethnic relations with Filipino Americans, alluding to the enduring Filipino familial and co‐ethnic ties that permeate their lives, suggesting that ethnic identity depends on social context (Nagel, 1994; Schimmele & Wu, 2015). Though they are dispersed throughout California, the largest number of Filipino immigrants resides in Southern California (McNamara & Batalova, 2015), fostering considerable opportunities for creating and establishing networks of familial and social group relationships that reinforce feelings of belonging and affiliation.

Moreover, in these geographic concentrations of Filipinos or enclaves, restaurants offer readily available ethnic food, and the residents speak native dialects as well as continue traditional cultural values and practices. Our self‐identification findings concur with other studies showing that the presence of relatively high levels of co‐ethnic geographic concentrations strengthen the retention of ethnic affiliation with immigrants from the country of origin and their descendants (Constant, Schuller, & Zimmerman, 2013).

### 4.3 Strengths and limitations and Directions for future Research

This study represents the first empirical report on the use of ASASFA in adult U.S.‐born FAs. In contrast, most acculturation studies on U.S.‐born FAs have focused on young children and adolescents (Espiritu & Wolf, 2001; Portes & Rambaut, 2006) using nonethnic specific acculturation measures. Our study revealed the biculturalism of adult U.S.‐born FAs: their shift to English in language use and preference and the simultaneous preference for co‐ethnic Filipinos in ethnic social relations.

This study has several limitations. The first limitation centers on the convenience sample of U.S.‐born FAs, mostly comprised of young and middle adults from the Greater Los Angeles Area in Southern California. Given that majority of Filipino immigrants reside in this area in Southern California (McNamara & Batalova, 2015), their presence facilitates the use of Philippine languages and interaction with Filipino immigrants, infusing a bias toward the second generation's retention and maintenance of their heritage culture. Hence, the generalizability of the results applies to U.S.‐born young and middle adults to the extent that they live in similar cultural/environmental contexts. A similar study can be conducted in other states and regions that have lesser concentrations of Filipino immigrants to obtain comparative acculturation data.

The second limitation stems from the study's use of a cross‐sectional research design, using a self‐report questionnaire. Respondents could have answered the ASASFA questions in a socially desirable manner. Also, this research design precludes causality. However, the results of studies on the children of post‐1965 immigrants consistently support the study's findings on their acculturation levels and the factors that predict their language use and preference and preference for ethnic social relations (Espiritu & Wolf, 2001; Portes & Rambaut, 2006). Future studies aiming to capture a more comprehensive scope of the acculturation of U.S.‐born FAs must include additional measures of cultural values, beliefs, practices, and ethnic identity. Furthermore, to apprehend the dynamic process of acculturation, a longitudinal design including a qualitative approach can discover the contextual aspects shaping the process, and the sources and amount of acculturative change in U.S.‐born adults (Fuligni, 2001; Ozer, 2013).

Third, the study did not assess the acculturation level of the sample's parents. It is possible, in particular, that more acculturated immigrant parents may have encouraged their children to use the English language rather than the heritage language at home to heighten the children's academic achievement and progression.

The fourth limitation relates to the use of only one question related to self‐identification, limiting the capture of the multidimensionality of the concept of ethnic identity that includes not only self‐identification but also group attachment, in‐group attitudes, and ethnic involvement (Schimmele & Wu, 2015). These dimensions reflect not only self‐ascription but also ascription by others—and they emerge as a result of intergroup relations (Schimmele & Wu, 2015). To capture these dimensions and the changeableness of ethnic identity across contexts would require a longer psychometrically valid measure.

### 4.4 Conclusion

This study shows that ASAFA demonstrates acceptable reliabilities and construct validity by Cronbach's alpha, EFA, and parallel analysis. ASASFA captures the within group differences among U.S.‐born FA adults in two common aspects of acculturation: language use and preference and preference in ethnic social relations. Hence, as an 11‐item acculturation scale, it provides a parsimonious tool for future studies focusing on FA health that treat acculturation as a variable. The availability of a valid and parsimonious tool supports its use when lengthy bidirectional acculturation measures become impractical in studies of immigrant health.
